# Retraction: Significance of B10 cell in patients with thymoma complicated with myasthenia gravis

**DOI:** 10.18632/oncotarget.28160

**Published:** 2023-04-10

**Authors:** Yang Lu, Fanjie Meng, Yang Yang, Lan Li, Donghao Wang, Yuantao Cui, Shangwen Dong, Wanhua Wang

**Affiliations:** ^1^Department of Intensive Care Unit, Tianjin Medical University Cancer Institute and Hospital, National Clinical Research Center for Cancer, Key Laboratory of Cancer Prevention and Therapy, Tianjin, Tianjin’s Clinical Research Center for Cancer, Tianjin, 300060, China; ^2^Department of Cardiothoracic Surgery, Tianjin Medical University General Hospital, Tianjin, 300052, China; ^3^Department of Anesthesia, Tianjin Medical University Cancer Institute and Hospital, National Clinical Research Center for Cancer, Key Laboratory of Cancer Prevention and Therapy, Tianjin, Tianjin’s Clinical Research Center for Cancer, Tianjin, 300060, China; ^4^Key Laboratory of Pharmacology of Traditional Chinese Medicine Formulae, Ministry of Education, Tianjin University of Traditional Chinese Medicine, Tianjin, 300193, China; ^*^These authors have contributed equally to this work and share first authorship

**This article has been retracted:** Oncotarget is retracting this article at the request of the authors. The accuracy and authenticity of the data need further analysis. Figure 2 contains incorrect flow cytometry images, and Figures 1 and 3 contain inaccurate data based on flawed patient criteria. As a result, the conclusions of this paper can no longer be verified. All authors, as well as the oversight committee of Tianjin Medical University, have agreed to the retraction of this paper.

Original article: Oncotarget. 2017; 8:73774–73786. 73774-73786. https://doi.org/10.18632/oncotarget.17908

